# Supplementary figures and images for: A taxonomic and molecular survey of the pteridophytes of the Nectandra Cloud Forest Reserve, Costa Rica
Source: PLoS One. 2020 Nov 18;15(11):e0241231. doi: 10.1371/journal.pone.0241231 (PMC7673574; doi:10.1371/journal.pone.0241231)

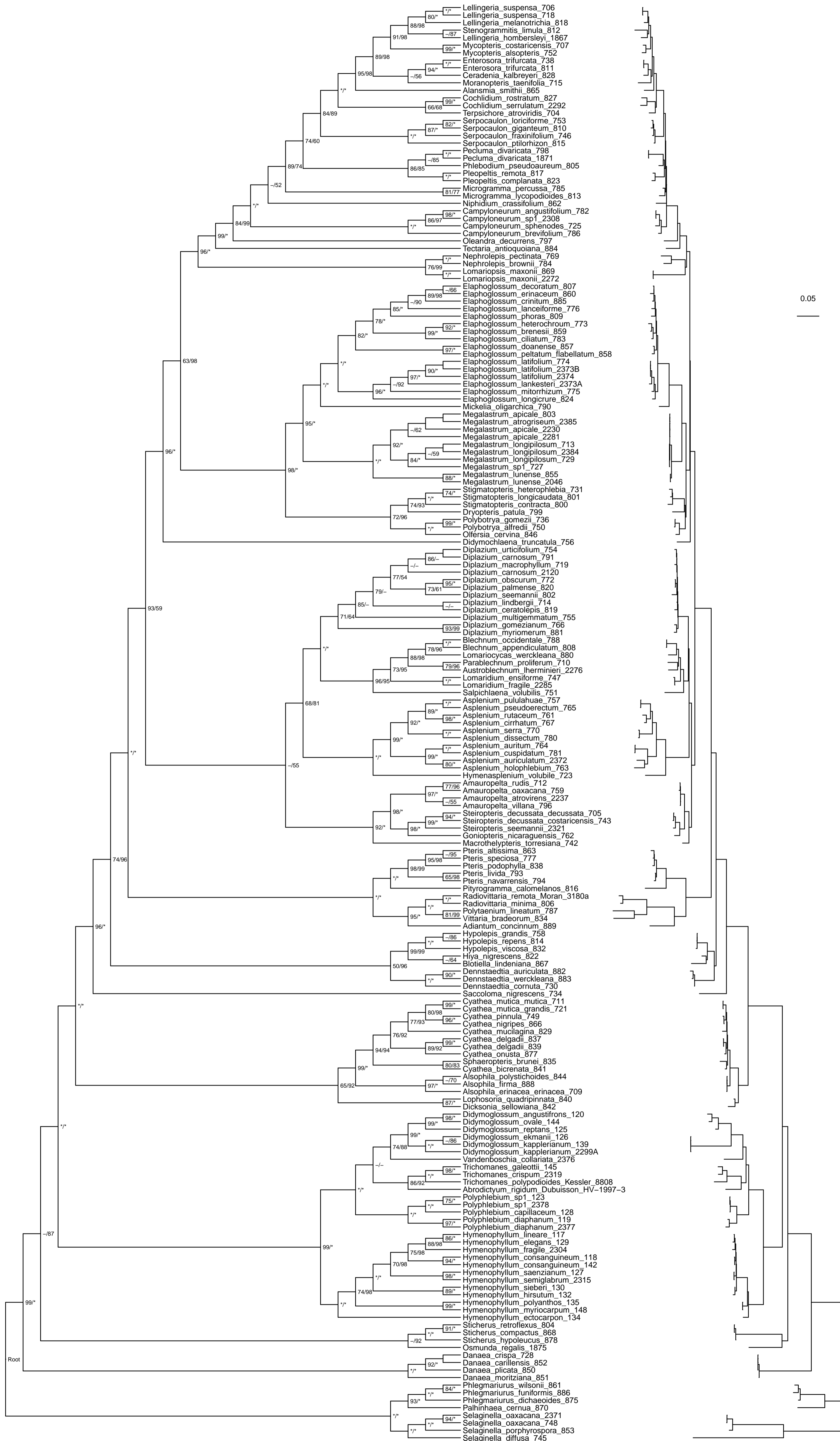

Supplement: S1 Fig — Tree rooted on lycophytes. Numbers at nodes indicate SH-aLRT support (%)/UFboot support (%); values less than 50% shown with “-”; values of 100% shown with “*”; completely blank nodes indicate identical sequences. For phylogram on right side, scale bar shows expected number of changes per site. Numbers after species name are J. H. Nitta specimen collection numbers except for Abrodictyum rigidum (J.-Y. Dubuisson HV 1997–3, Venezuela), Trichomanes polypodiodes (M. Kessler 8808, Bolivia), and Radiovittaria remota (R. Moran 3180a, Costa Rica), which could not be sequenced successfully, so GenBank sequences were used instead (accessions AY095108, AY175795, and U21289, respectively). All J. H. Nitta specimens from Nectandra. (PDF) [file pone.0241231.s001.pdf]

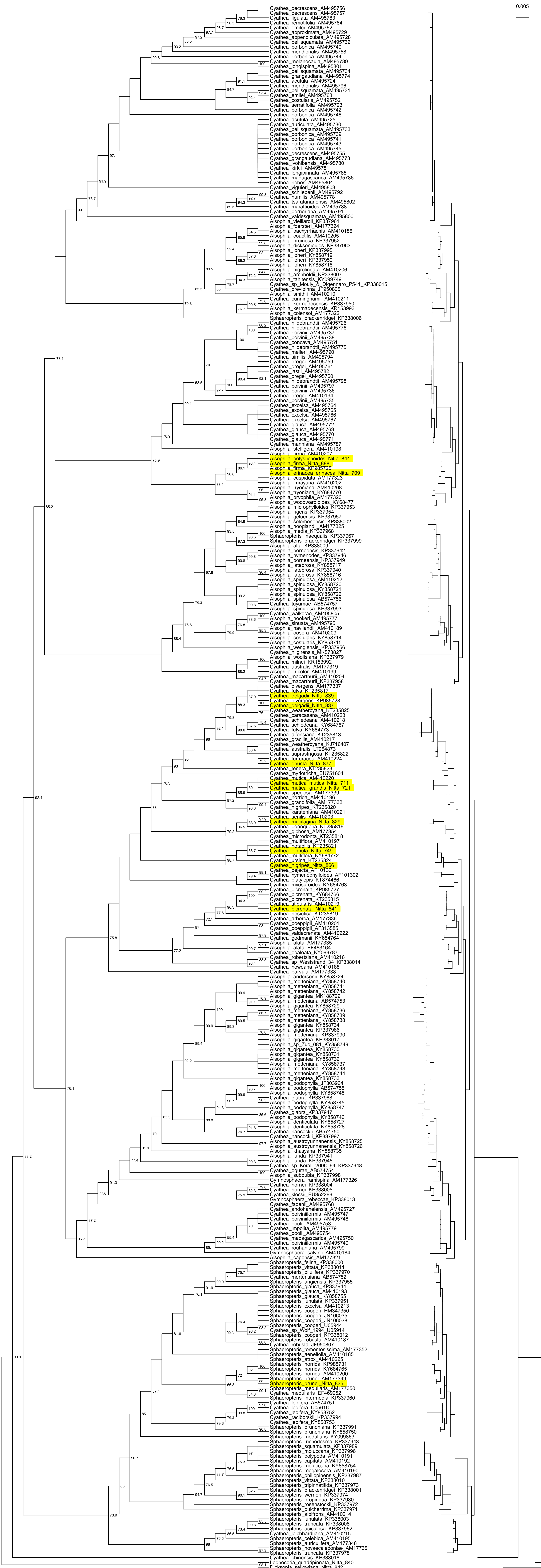

Supplement: S2 Fig — Tree rooted on species from Nectandra in family Dicksoniaceae. Numbers at nodes indicate local support values computed with the Shimodaira-Hasegawa test; values less than 50% not shown. For phylogram on right side, scale bar shows expected number of changes per site. Numbers after species names are GenBank accession numbers for sequences downloaded from GenBank or J. H. Nitta specimen collection numbers for sequences newly obtained by this study. Newly obtained ingroup sequences highlighted in yellow. (PDF) [file pone.0241231.s002.pdf]
