# Supplementary material for: A taxonomic and molecular survey of the pteridophytes of the Nectandra Cloud Forest Reserve, Costa Rica
Source: PLoS One. 2020 Nov 18;15(11):e0241231. doi: 10.1371/journal.pone.0241231 (PMC7673574; doi:10.1371/journal.pone.0241231)

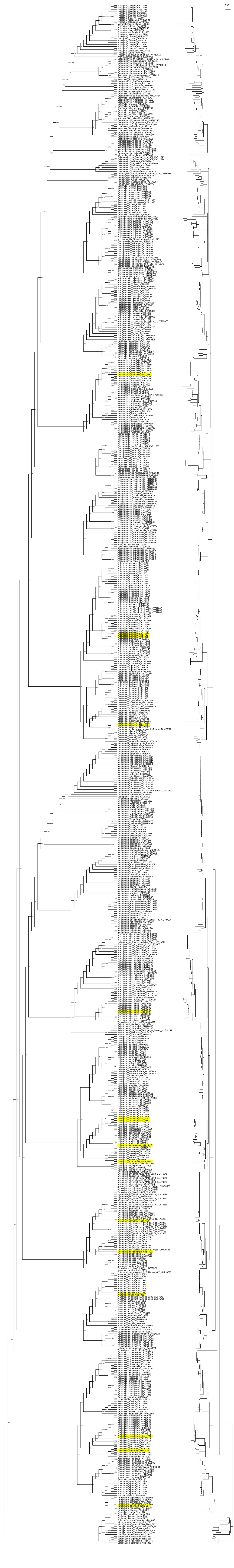

Supplement: S3 Fig — Tree rooted on species from Nectandra in subfamily Polypodioideae. Numbers at nodes indicate local support values computed with the Shimodaira-Hasegawa test; values less than 50% not shown. For phylogram on right side, scale bar shows expected number of changes per site. Numbers after species names are GenBank accession numbers for sequences downloaded from GenBank or J. H. Nitta specimen collection numbers for sequences newly obtained by this study. Newly obtained ingroup sequences highlighted in yellow. (PDF) [file pone.0241231.s003.pdf]
